# Repurposing Anticancer Drugs Targeting the MAPK/ERK Signaling Pathway for the Treatment of Respiratory Virus Infections

**DOI:** 10.3390/ijms25136946

**Published:** 2024-06-25

**Authors:** Yuchen Liu, Zhijun Luo

**Affiliations:** Medical Department, Queen Mary School, Nanchang University, Nanchang 330031, China; yuchen.liu@se21.qmul.ac.uk

**Keywords:** respiratory virus, SARS-CoV, COVID-19, influenza, infections, MAPK/ERK pathway, MEK inhibitors

## Abstract

Respiratory virus infections remain a significant challenge to human health and the social economy. The symptoms range from mild rhinitis and nasal congestion to severe lower respiratory tract dysfunction and even mortality. The efficacy of therapeutic drugs targeting respiratory viruses varies, depending upon infection time and the drug resistance engendered by a high frequency of viral genome mutations, necessitating the development of new strategies. The MAPK/ERK pathway that was well delineated in the 1980s represents a classical signaling cascade, essential for cell proliferation, survival, and differentiation. Since this pathway is constitutively activated in many cancers by oncogenes, several drugs inhibiting Raf/MEK/ERK have been developed and currently used in anticancer treatment. Two decades ago, it was reported that viruses such as HIV and influenza viruses could exploit the host cellular MAPK/ERK pathway for their replication. Thus, it would be feasible to repurpose this category of the pathway inhibitors for the treatment of respiratory viral infections. The advantage is that the host genes are not easy to mutate such that the drug resistance rarely occurs during short-period treatment of viruses. Therefore, in this review we will summarize the research progress on the role of the MAPK/ERK pathway in respiratory virus amplification and discuss the potential of the pathway inhibitors (MEK inhibitors) in the treatment of respiratory viral infections.

## 1. Introduction

Respiratory viral infections that occur upon virus invasion of the respiratory mucosa pose a significant threat to humans and exert a substantial impact on global health [1]. Respiratory viruses, comprising diverse viral families, lack a specific viral taxonomy; their genomic structures, susceptible populations, severity of diseases, seasonality, transmissibility, and modes of transmission vary across viral strains [2]. The seasonal transmission of influenza A virus, spanning from September 2019 to February 2020, resulted in a minimum of 34 million cases of sickness and 20,000 deaths [3]. An epidemic of severe acute respiratory syndrome (SARS) caused by the β-coronavirus (SARS-CoV) emerged extensively in November 2002, spreading in 29 countries, resulting in approximately 8000 infections and 774 fatalities [3]. In the 2020 pandemic, the coronavirus 2019 (SARS-CoV-2) disease (COVID-19) caused more than 1.8 million deaths [4]. In addition, adenoviruses among at least nine other distinct viruses have been described to date as common pathogens causing respiratory tract infections [5,6].

All these viral infections start from the respiratory tract and could invade the whole body, causing respiratory and systemic dysfunctions. Respiratory viruses can spread via four modes—direct contact, indirect contact, droplets, and aerosols—and cause a spectrum of diseases ranging from mild respiratory illnesses to acute pneumonia and even respiratory failure, depending on the timing of infection and viral load [2,3]. Mild symptoms include rhinitis, nasal congestion, sore throat arising from upper respiratory tract infection by rhinovirus, adenovirus, and human coronaviruses that are typically confined to the upper respiratory tract [1]. Some viruses may lead to severe lower respiratory tract infections, such as human respiratory syncytial virus (HRSV), which can move to the lower respiratory tract after an incubation period, causing pneumonia, bronchiolitis, tracheobronchitis, and croup [6]. The infection of human parainfluenza viruses (HPIVs) generate symptoms similar to those of HRSV, where viral infection of epithelial cells triggers an amplified inflammatory response, leading to mononuclear infiltration of the interstitium, epithelial necrosis, and exudation of inflammatory mediators into the alveoli. This could eventually result in pulmonary consolidation with complications of febrile upper respiratory infections and otitis media, occurring in two-thirds of infected children. The respiratory viral infections induce epithelial damage and immune dysregulation, which can increase bacterial colonization in the respiratory tract (upper and lower), secondary bacterial infection, and also alter the gut microbiota by inducing a loss of appetite [7,8]. The respiratory viral infections disrupt the integrity of the airway epithelium, induce cytokine production, and may even disseminate systemically. Severe viral infections can lead to critical illness, including acute respiratory failure; for example, coronaviruses can trigger cytokine storms resulting from excessive immune responses, manifesting severe pulmonary pathology and systemic reactions, which are particularly pronounced in vulnerable populations [3,9].

Many respiratory viral infections are self-limiting, meaning that patients recover through the body’s immune system without medication. However, if defensive immunity is compromised or new variants emerge, illness could be severe, and the infection even becomes epidemic. Several antiviral drugs (e.g., Molnupiravir, Remdesivir, Nirmatrelvir, and Ritonavir) have been developed against viral infection and appear to be effective to control progression of the disease. However, drugs directly targeting viruses can easily render them resistant, especially facing an epidemic and pandemic, as the replication of viruses is very quick, which offers an advantage of natural selection for drug-resistant variants. Therefore, a wise strategy might be to target the host machinery that is necessary for viral replication as an alternative approach. In fact, previous studies have shown that many viruses hijack host kinases for their amplification and that inhibition of these kinases could lead to suppression of virus production [10,11,12,13,14]. As MEK inhibitors are already clinically available in cancer therapy, it would be an intriguing idea to repurpose this category of anticancer drugs to treat the respiratory viral infections and prevent their spread. Thus, this review focuses on the utility of the MAPK/ERK pathway by respiratory viruses and discusses the potential of MEK inhibitors in the treatment of the viral infections.

## 2. The Roles of the MAPK/ERK Pathway in Viral Infections

**Figure 1:**
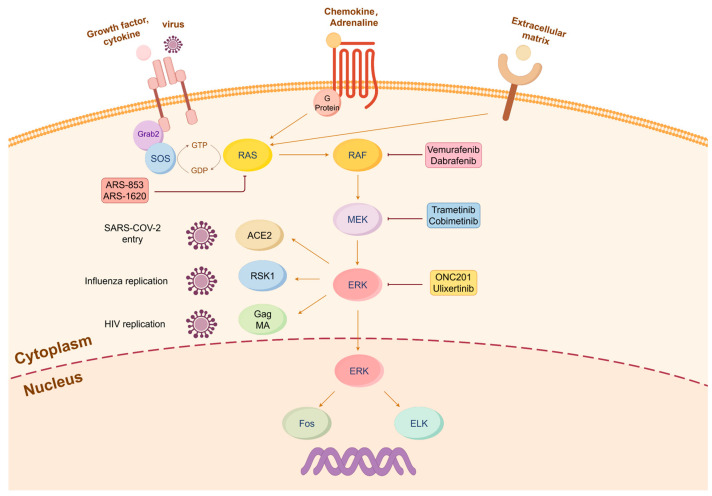
The role of the MAPK/ERK pathway in virus replication. The canonical MAPK/ERK cascade is triggered by the binding of extracellular ligands (e.g., growth factor or viral envelop proteins), chemokine, and extracellular matrix. The activated MAPK/ERK pathway is exploited by various viruses to sustain their self-replication. For instance, RSK1 plays a pivotal role in the nuclear export of vRNP during influenza virus replication, Gag MA protein facilitates the replication of the HIV reverse transcription complex, ACE2 receptor serves as the key receptor for SARS-CoV-2 infection, and the c-fos transcription factor downstream of ERK is hijacked for the replication of HSV2 (herpes simplex virus 2). The pathway inhibitors for cancer therapy that can be possible to repurpose are highlighted.

The MAPK/ERK pathway is the first well-characterized mitogenic regulatory hub, mainly consisting of three-tiered serine/threonine kinases, Raf, MEK, and ERK, which plays a pivotal role in cell proliferation, differentiation, survival, and development [15]. Extracellular ligands bind to and activate receptor tyrosine kinase (RTK) at the plasma membrane to trigger the canonical signaling cascade. Upon activation, the tyrosine-phosphorylated receptor transmits the signal through growth factor receptor-bound protein 2 (Grb2) and son of sevenless (SOS) to activate Ras, which is a small GTPase. In the resting state, RAS is GDP-bound. In response to growth factors, SOS, a guanine nucleotide exchange factor, promotes the exchange of GDP with GTP on Ras. GTP-loaded Ras induces activation of Raf, which causes sequential phosphorylation and activation of MEK1/2 and ERK1/2. Most of the functions of the MAPK/ERK pathway are executed by ERK1/2; namely, they have a broad spectrum of substrates (Figure 1) [16,17]. In addition to the response to the ligands that activates RTK, the MAPK/ERK pathway can be activated by many other signals such as seven transmembrane receptors coupled with trimeric G protein coupled with receptors, cytokines, chemokines, extracellular matrix, stress, etc. [18,19]. The dysregulation of the MAPK/ERK pathway is associated with neoplasm, where it enhances cancer cell proliferation, invasion, and metastasis [20].

As almost all of oncogenes directly or indirectly lead to activation of the MAPK/ERK pathway, great efforts have been vested to develop drugs to interfere with this pathway. Sobimetinib, trametinib, binimetinib, and selumetinib are the four drugs currently approved as MEK inhibitors by the FDA for use in the treatment of neurofibromatosis type 1 in children and other cancers with constitutively active MEK and ERK [15,21]. Other combinations of B-Raf and MEK inhibitors (e.g., vemurafenib and cobimetinib, encorafenib and binimetinib, and dabrafenib and trametinib) are used to treat melanomas with B-Raf mutations and the constitutively active MAPK/ERK pathway [22].

In addition to the regulatory role in cell growth, the MEK/ERK pathway is also widely exploited in viral replication [23,24,25,26]. In HIV infection, phosphorylation of the viral protein Gag MA facilitates membrane dissociation of the reverse transcription complex, leading to its translocation into the nucleus to promote viral replication [12]. Treatment with the MEK inhibitor trametinib suppresses Treg cells to enhance the host’s anti-HIV capability and binding capacity, while also downregulating pro-inflammatory factors [27]. Thus, the inhibitors for all components of the MAPK/ERK pathway that are exploited by viruses can possibly be repurposed for the treatment of viral infections, as highlighted in Figure 1 (for detailed pathway inhibitors, please refer to [18]). Since the B-Raf and MEK inhibitors are so far the most successful in clinical use of anticancer therapy while investigations of B-raf inhibitors are mostly focused on B-Raf V600E mutation, it is feasible to repurpose the MEK inhibitors for antiviral infections. The MEK inhibitors PD98059 and U0126 were the first to show the blockade of virus replication [23,24,25,26,28]. U0126 exhibits a strong synergistic effect when used in combination with the anti-CMV drug ganciclovir (GCV) [29]. Although MEK inhibitors as anticancer drugs have side effects such as rash and fatigue, their use in antiviral therapy has an advantage of shorter cycles and smaller dosages [30]. Thus, the treatment of viral infection with MEK inhibitors sounds to be an implementable option.

### 2.1. Influenza Virus

Influenza viruses contain a negative single-stranded RNA genome composed of seven or eight RNA nucleic acid segments, and utilize RNA-dependent RNA polymerase (RdRp), which binds to the 5′ and 3′ ends of the RNA, along with PB1, PB2, and PA proteins, to orchestrate synthesis of oligomeric viral nucleoprotein (NP), a key component of the viral ribonucleoprotein (vRNP) complexes. vRNP is essential for the viral replication cycle [31]. The interaction between the cell surface receptor and hemagglutinin (HA) glycoproteins facilitates the binding and internalization of influenza virus into target cells, followed by the low pH environment within the endosome to trigger the opening of viral integral membrane protein M2 and the entering of the proton into the virus [32,33,34]. Acidification induces fusion of the endosome and HA, releasing vRNP into the cytoplasm and entering the nucleus, where subsequent two-step replication takes place by utilizing cRNA as intermediates. During the late stages of viral infection, the viral proteins M1 and NEP localized in the nucleus facilitate the export of vRNP into the cytoplasm, where vRNP is encapsulated within the viral envelope composed of the host membrane and viral transmembrane proteins, resulting in budding and the generation of viral particles [32]. Over half of influenza viral infections are asymptomatic, while the rest have common symptoms including high fever, rhinitis, cough, and headache, and some can develop severe symptoms arising from infection-induced cytokine storms, leading to primary viral pneumonia, acute respiratory distress syndrome (ARDS), and pulmonary edema, with secondary bacterial pneumonia possible during the convalescent phase of influenza [35].

**Figure 2:**
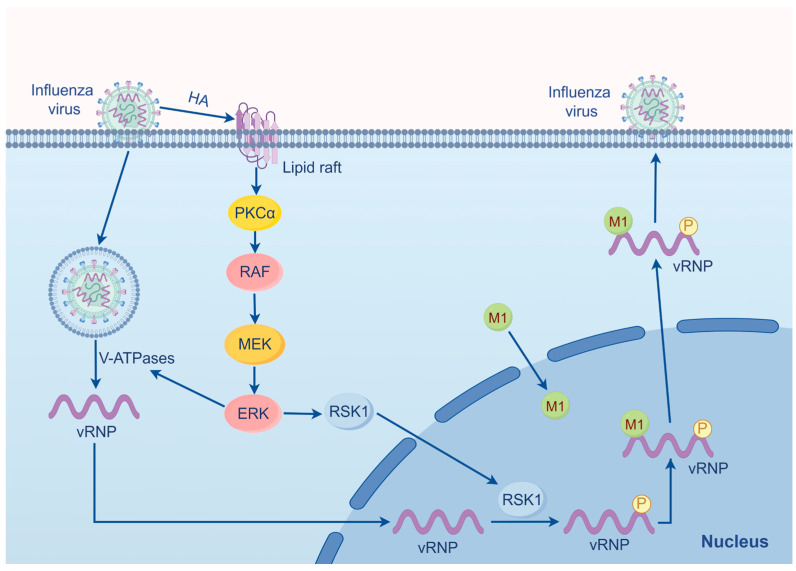
The process by which influenza virus utilizes the MAPK/ERK pathway for replication. In the early stage of infection, HA of the virus binds to lipid rafts to activate PKCα, subsequently activating the RAF/MEK/ERK pathway. Activated ERK enhances V-ATPase activity, leading to endosomal acidification, viral dissociation from the membrane of endosomes, and the release of vRNP, which enters the nucleus. In the late stage, RSK1 activated by ERK translocates to the nucleus, where it phosphorylates vRNP. The phosphorylated vRNP binds to newly synthesized M1 protein, translocates to the cytoplasm, and combines with structural proteins to assemble new viral particles.

The influenza virus infection involves four subfamilies of MAPK, with the MAPK/ERK cascade contributing the most [36]. In both influenza virus A and B infection, the activation of the MEK/ERK pathway occurs in a biphasic manner detected at both early and late phases of infection (Figure 2) [37]. At the early phase, influenza activates MAPK/ERK, leading to an increase in the activity of Vacuolar H-ATPases (V-ATPases), thereby creating an acidic environment in the endosome that facilitates the nuclear import of vRNP [38]. In the late stage of infection, the outcome of the MAPK/ERK activation primarily stimulates the nuclear export of vRNP. Research indicates that application of the MEK inhibitor U0126 in an influenza A infection model impairs the function of the nuclear export protein (NEP/NS2) and thus obstructs vRNP export, while not affecting viral RNA or HA and NP synthesis [11,36,39]. Virus-induced ERK activation, distinct from other signaling pathways, operates independently of dsRNA and viral replicative intermediates [40]. The viral polymerase PB1 regulates HA synthesis, whereby HA binding to lipid rafts at the cell membrane that activates PKCα, leading to the activation of Raf/MEK/ERK. As a result, cytoplasmic localization of vRNP is increased and the viral titer elevated [36,38,41]. During the entry of the virus into the host cells, the platelet-derived growth factor receptor also activates the MAPK/ERK signaling pathway [42].

Tyr132 of matrix protein (M1) is a phosphorylated site on the influenza A virus that drives the nuclear import of M1 and promotes viral replication in the late phase of the infection [43]. The MAPK/ERK pathway assists vRNP in exporting from the nucleus into the cytoplasm by promoting the association of vRNP with M1 on chromatin [37]. The NP of the vRNP complex is phosphorylated at serine residues S269 and S392 by RSK1 downstream of ERK, as evidenced by the abrogation of the phosphorylation at these two sites following treatment with the MEK inhibitor CI-1040 [37]. RSK1 migrates into the nucleus, where NP is phosphorylated by RSK1. Upon phosphorylation, NP interacts with M1, facilitating the nuclear export of newly synthesized vRNP [37]. However, the nuclear output of vRNP depends on its binding with Crm1/exportin 1 on chromatin.

### 2.2. SARS-CoV-2

**Figure 3:**
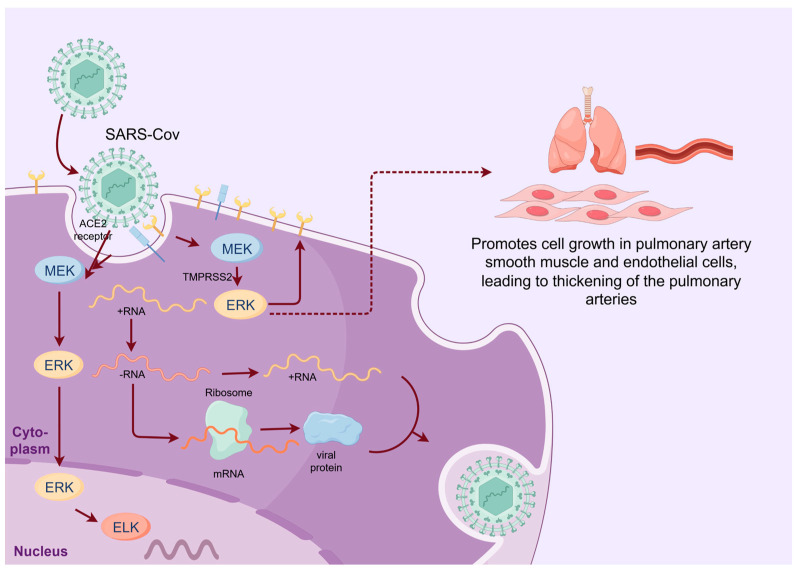
Utilization of the MAPK/ERK pathway by SARS-CoV-2. The entry of SARS-CoV-2 results in TMPRSS2-mediated cleavage of the S protein into S1 and S2 subunits. Binding of the S1 protein to the ACE2 receptor activates the MAPK/ERK pathway, leading to increased expression of ACE2 on the cell surface. The S2 protein promotes fusion of the virus with cellular membranes. Upon entry of the virus, the positive single-stranded RNA (+RNA) is released and transcribed into negative RNA (-RNA), which in turn serves as a template to synthesize positive RNA (+RNA) and mRNA. Finally, +RNA and viral proteins assembled as new virus particles. The activation of ERK can increase cell growth of pulmonary artery smooth muscles and endothelial cells, leading to thickening of the pulmonary arteries. In PDCoV replication, at early stage, ERK translocates into the nucleus and phosphorylates EIK-1.

SARS-CoV-2 is a positive single-stranded RNA beta-coronavirus encoding four non-structural proteins: 3-chymotrypsin-like protease, papain-like protease, helicase, and RNA-dependent RNA polymerase (Figure 3) [44]. Following infection with SARS-CoV-2, 80% of mild cases exhibit symptoms including a fever, cough, sore throat, anosmia, headache, and body aches [1]. Moderate cases come across with viral infection of the lower respiratory tract, while severe infection triggers a cytokine storm characterized by the systemic release of cytokines, hypotension, and acute respiratory distress syndrome (ARDS) [1]. Entry of SARS-CoV-2 into the host cells relies on host ACE2, a SARS-CoV-2 receptor, and the host cell membrane-associated transmembrane protease, serine 2 (TMPRSS2) [44]. The spike protein is activated following cleavage at the S1/S2 site by the serine protease TMPRSS2, and then the S1 portion binds to the ACE2 while S2 drives fusion of the virus with the cell membrane [45]. Upon entry into the host cells, the coronavirus genome is released into the cytoplasm, where positive-sense RNA is transcribed to produce antisense RNA [46]. The full-length negative-sense genome serves as a template for the synthesis of both positive-sense RNA and mRNA. The newly synthesized genomes then serve as templates to generate additional non-structural proteins (NSPs) and replication, and transcription complexes (RTCs) are packaged into new virus particles [46].

The replication of SARS-CoV-2 exploits the MAPK/ERK pathway but is less dependent than influenza virus [47]. SARS-CoV-2 infection triggers early monophasic activation of the MAPK/ERK signaling pathway, which plays an important role in the early stage of the life cycle, engaging the TMPRSS2-mediated cell surface entry mechanism [48]. The S1 subunit also triggers phosphorylation of MEK and activation of the downstream pathway 49]. The activated ERK modulates ACE2 expression levels. Thus, MEK inhibitors exert a negative influence on ACE2 expression, thereby mitigating early infection [6]. On the other hand, the activated MEK/ERK pathway promotes cell growth in pulmonary artery smooth muscle and endothelial cells, leading to thickening of the pulmonary arteries, which was observed in lung sections from patients who died of COVID-19, in contrast to patients who died of influenza virus [49]. In addition, the S protein also upregulates expression of COX-2, an inflammatory enzyme, which activates PKCα upstream of the Raf [50].

Other coronaviruses such as infectious bronchitis virus (IBV) also exploit ERK to prolong cell survival, so as to promote efficient IBV replication [28]. ERK upregulates Mcl-1 and Bcl-2, thereby inhibiting cell apoptosis and promoting viral replication [50]. Counteracting this, ERK also upregulates phosphatase DUSP6 to negatively regulate ERK activity, impairing viral replication and promoting cell apoptosis, thus mediating cellular protective mechanisms. In Porcine deltacoronavirus (PDCoV) infection, ERK activation is monophasic at an early phase during the viral entry into cells, where both viral and cellular cholesterol play a crucial role. However, the ERK activation is not dependent on viral replication [51]. Upon early, intense but transient activation, ERK translocates into the nucleus and phosphorylates EIK-1, modulating viral replication.

## 3. The MEK/ERK Pathway Serves as a Novel Antiviral Target

Given that a large number of studies have proven that many viruses engage the MAPK/ERK pathway in their replication, such as influenza viruses, SARS-CoV-2, and murine coronavirus mouse hepatitis virus (MHV), this pathway could be a promising target for virus prevention and treatment. The application of MEK inhibitors in blocking the replication of various viruses has been extensively investigated and parts of them are listed in Table 1. The MAPK/ERK signaling pathway participates in infection-specific regulation by MERS-CoV [28]. Thus, kinase inhibitors targeting the ERK1/2 signaling pathway, such as selumetinib and trametinib, when administered before or after infection, effectively suppress the majority of MERS-CoV infections. CI-1040, a clinically effective MEK inhibitor in cancer therapy, inhibits activation of RSK1, leading to impaired vRNP output and reduction in the virus titers [37]. Borna disease virus (BoDV) is the only known virus, apart from influenza virus, with a nuclear phase, whose replication is also impaired by MEK inhibitors [10].

The critical factor that transitions respiratory viral infectious diseases into severe, even lethal cases is the cytokine storm, where excessive pro-inflammatory factors are produced [6,48]. In addition to directly impeding viral replication, MEK inhibitors can mitigate the release of pro-inflammatory factors in patients by gating them within a reasonable range. In SARS-CoV-2, MEK inhibitors stimulate the activity of natural killer cells (but not T cells) and can also attenuate viral infectivity by downregulating the expression of ACE2 [6].

Quaglino et al. have observed that during the outbreak of COVID-19, 67 advanced melanoma patients in Dermatology Clinic, University of Turin, Italy, were treated by targeted therapy (58 with dabrafenib/trametinib, 6 with vemurafenib/cobimetinib, and 3 with encorafenib/binimetinib). None of them tested positive on SARS-2 [63]. Whether the negative test results are due to the reduced contact of the patients with infected people or the inhibition of MEK activity warrants further investigation. If the finding is attributable to the use of Braf and MEK inhibitors, it would be direct evidence that the MEK inhibitors can be used for the treatment of viral infections.

MEK inhibitors not only exhibit standalone efficacy in impairing viral replication, but also demonstrate synergistic effects when used in combination with other antiviral agents. Zapnometinib, an active metabolite of CI-1040, has been shown to directly inhibit virus replication when used alone, and also to exhibit a significant synergistic effect, improving the efficacy and alleviating adverse reactions when used in combination with Direct-Acting Antivirals (DAA) such as Molnupiravir, Remdesivir, Nirmatrelvir, and Ritonavir [64]. Remdesivir and MEK inhibitors that are administered in doses to modulate SARS-CoV-2 infection, immune responses, and cytokine levels manifest no toxicity to normal cells and cancer cells [6]. A previous investigation has demonstrated that treatment with various MEK inhibitor (MEKi) compounds such as VS-6766 reduces ACE2 cellular expression and inhibits pseudovirus infection in normal human small airway epithelial (HSAE) cells [65]. GLPG-0187, an integrin/TGF-β1 inhibitor, exhibits enhanced inhibitory effects on pseudovirus infection when HSAE cells are pre-treated with the MEK inhibitor (MEKi) VS-6766.

## 4. The Clinical Development of MEK Inhibitors for Antivirus

CI-1040 (PD 184352) is an orally active, highly specific, non-ATP-competing small molecule inhibitor of MEK with an IC50 value of 17 nM for MEK1 and the first anticancer drug to enter clinical trials with the advantage of low toxicity [56,66]. The most common toxic reactions are diarrhea, weakness, rash, nausea, and vomiting, with a small number of patients experiencing grade III adverse reactions [67]. CI-1040 has been shown to inhibit a variety of cancers, such as neuroblastoma, pancreatic, colon, breast, and non-small-cell lung carcinoma [68]. Similar to other protein kinase inhibitors, CI-1040 showed stronger inhibition of non-phosphorylated MEK than activated phosphorylated MEK1 [69]. Clinical studies have shown that CI-1040 exerts an antiviral effect. In influenza virus replication, CI-1040 showed an obvious inhibition of the phosphorylation of NP such that it lost the ability of nuclear export and thus the replication of the influenza virus is inhibited [37].

CI-1040 has a faster clearance rate in plasma, even administrated with high doses, while zapnometinib (also known as PD-0184264 or ATR-002) maintains a much higher plasma concentration [62]. Although studies have shown that the in vitro concentration of zapnometinib is more than 10-times that of CI-1040 to achieve the same inhibitory effect, only one-sixth of the dose of CI-1040 can significantly reduce viral titer in animal experiments [70]. In mice, it was shown that zapnometinib is rapidly absorbed and detectable even after 168 h, demonstrating that the drug has good bioavailability and it is mainly distributed in the circulatory system and visceral tissues [71]. The maximum plasma concentration of zapnometinib is reached between 2 and 4 h after administration, rapidly excreted, and significantly cleared from the plasma after 24 h, most of which is detected in the feces, primally through feces and secondly through urine [71,72]. MEK inhibition can be up to 50 percent when administered at the highest doses, and the inhibitory effect on MEK remains even after the plasma concentration of zapnometinib has decreased or even become nondetectable [64]. In a double-blind phase II trial of hospitalized adults with COVID-19, zapnometinib demonstrated a good safety profile with a low incidence of treatment-emergent adverse effects (TEAEs) [73].

## 5. Discussion

The MAPK/ERK cascade constitutes a vital intracellular signaling pathway governing essential cellular processes such as proliferation, survival, and differentiation [15]. The widespread exploitation of this pathway by cancer cells is not surprising, given its pivotal role and its aberrant activation in over 30% of human cancers containing Ras mutations [30] and nearly all cases of cutaneous melanoma [21]. Recent studies have shown that many viruses hijack the host MAPK/ERK pathway for replication, including influenza, SARS-CoV-2, enteroviruses, vaccinia virus, and yellow fever virus, although downstream targets and the precise mechanisms vary [24,25,37]. Viruses continue to pose a significant threat to human life, as exemplified by highly pathogenic respiratory viruses such as influenza viruses and the recently emerged SARS-CoV-2 [10]. However, the efficacy of antiviral drugs is not as robust as anticipated inasmuch as rapid replication of the viral genome engenders frequent mutations which favor generation of the drug-resistant variants [10]. Secondly, initial treatment after occurrence of symptoms in COVID-19 patients often misses the optimal opportunity, thus limiting the effectiveness of antiviral medications [72]. For influenza A virus, apart from prophylactic vaccination, only a few antiviral drugs have been approved. This underscores the urgent need for more effective antiviral medications to better control infections, particularly during early outbreaks when vaccines are not yet available [10,56].

Since many viruses exploit the host machinery for their replications, it is rational to repurpose clinically available drugs that target the host cell proteins essential for the replication of viruses as the side effects are known and the genes for host proteins are relatively resistant to mutations if the drugs used for a short period of time. The MAPK/ERK pathway is frequently exploited by a variety of viruses during infection. Hence, MEK inhibitors (MEKIs) could serve as broad-spectrum antiviral drugs, with less possibility to cause mutations-induced drug resistance than DAA. Moreover, the combined use of MEK inhibitors (MEKIs) with DAAs can enhance the efficacy of antiviral medications, potentially by reducing the required DAA concentration and alleviating adverse reactions. Thus, the combination exhibits therapeutic effectiveness against viral variants [64]. Awareness in using MEK inhibitors for the treatment of viral infection includes whether they affect the replication cycle of normal cells and have side effects: for example, those observed in clinical cancer therapy, such as rash, fatigue, vomiting, and diarrhea. However, the treatment duration for cancer typically spans two to three months and even longer, while viral infections are typically treated for only about a week. For respiratory viruses, an advantage is to administer drugs through mist spray.

Zapnometinib, which has entered clinical trials as a host-targeting antiviral drug (HTA), shows promising initial results in terms of biological safety and is presently under clinical development for the treatment of COVID-19 and influenza. This drug shows both antiviral and immunomodulatory effects [71,74]. In the presence of zapnometinib, the sensitivity of SARS-CoV-2 to antiviral drugs remains unaltered, with no change in susceptibility to zapnometinib observed in DAA-resistant strains, thereby providing therapeutic benefits even against viruses resistant to DAA [74]. In conclusion, MEK serves as a promising target for antiviral therapy, and the development of MEK inhibitors holds significant importance in combating emerging respiratory viral infections.

## Figures and Tables

**Table 1:** MEK inhibitors in studies of anticancer and antivirus.

No.	MEK Inhibitors	Structure	Anticancer	Antivirus (Preclinical)	References for Virus Studies
**1**	Trametinib	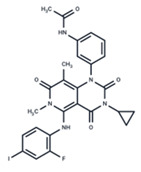	FDA approved	SARS-CoV-2 Influenza B HIV	[52,53,54]
**2**	Cobimetinib	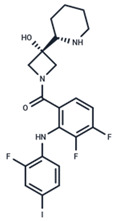	FDA approved	COVID-19 cytokine release	[55]
**3**	CI-1040	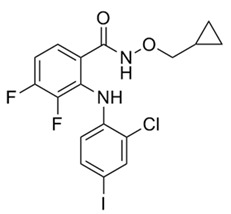	Phase II	Influenza	[56,57]
**4**	PD0325901	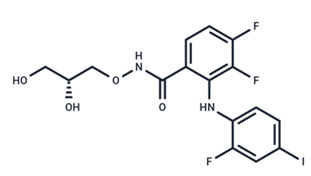	Phase II	Influenza A	[58]
**5**	Selumetinib	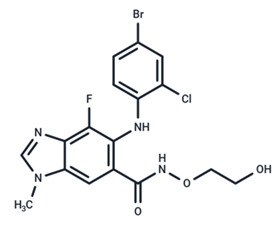	Phase III	SARS-CoV-2	[59]
**6**	MEK162	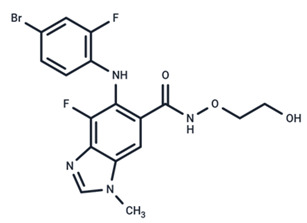	Phase III	No data	
**7**	AZD8330	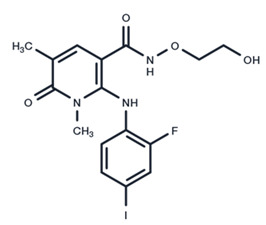	Phase I	Influenza A	[58]
**8**	TAK-733	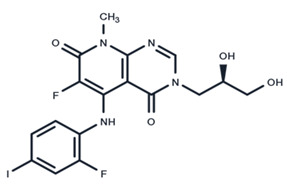	Phase I	No data	
**9**	GDC-0623	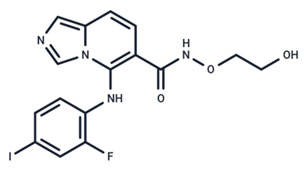	Phase I	No data	
**10**	Refametinib	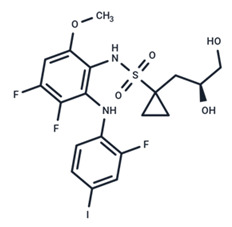	Phase II	No data	
**11**	Pimasertib	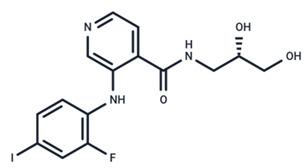	Phase II	Enteroviurs-A71	[60]
**12**	RO4987655	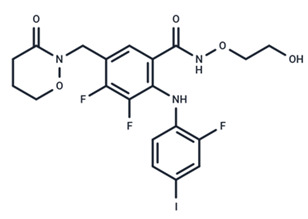	Phase I	No data	
**13**	RO5126766	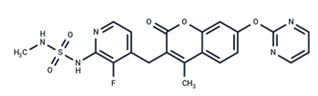	Phase I	No data	
**14**	HL-085	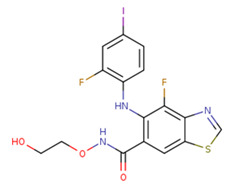	Phase I	No data	
**15**	CInQ-03	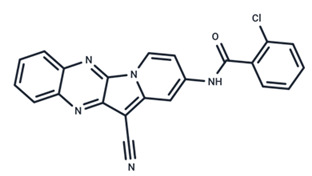	Preclinical	No data	
**16**	G-573	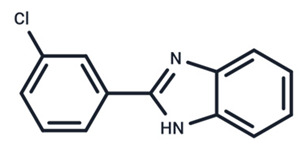	Preclinical	No data	
**17**	PD184161	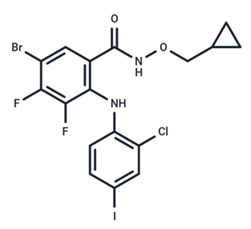	Preclinical	No data	
**18**	PD318088	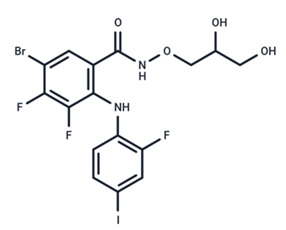	Preclinical	No data	
**19**	PD98059	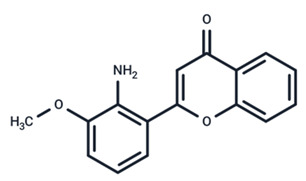	Preclinical	SARS-CoV-2	[61]
**20**	RO5068760	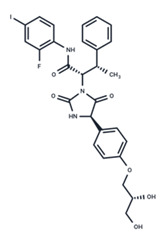	Preclinical	No data	
**21**	U0126	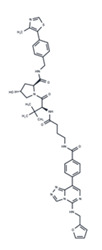	Preclinical	Influenza	[39]
**22**	SL327	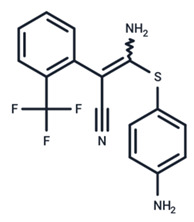	Preclinical	Enterovirus	[62]

## Data Availability

Not applicable.
